# Mortality of Women in Childbirth

**Published:** 1857-04

**Authors:** 


*Mortality of Women in Childbirth.*

In 1848, 61 mothers died to every 10,000 children born alive in Great Britain. Since that time the mortality has progressively declined as follows: 58, 55, 53, 52, 50, down to 47 in 1854.

This is a gratifying result, and there can be no doubt that, by further care and skill, and especially by training up a class of educated nurses, the deaths in child-birth may be largely reduced from their present high number, 3,009.

